# Single‐dose NEPA versus an aprepitant regimen for prevention of chemotherapy‐induced nausea and vomiting in patients receiving moderately emetogenic chemotherapy

**DOI:** 10.1002/cam4.6121

**Published:** 2023-08-03

**Authors:** Laurent Zelek, Rudolph Navari, Matti Aapro, Florian Scotté

**Affiliations:** ^1^ Hôpital Avicenne Bobigny France; ^2^ World Health Organization Cancer Care Program Birmingham Alabama USA; ^3^ Genolier Cancer Center Clinique de Genolier Genolier Switzerland; ^4^ Interdisciplinary Cancer Course Department Gustave Roussy Cancer Center Villejuif France

**Keywords:** aprepitant, CINV, NEPA, netupitant, palonosetron

## Abstract

**Introduction:**

Non‐inferiority of NEPA (fixed combination of NK_1_ receptor antagonist (RA), netupitant, and 5‐HT_3_RA, palonosetron) versus an aprepitant regimen was previously shown in a pragmatic study in patients receiving anthracycline cyclophosphamide (AC) and non‐AC moderately emetogenic chemotherapy (MEC). In the MEC group a numerically higher complete response (CR: no emesis, no rescue) rate was seen for NEPA during the overall 0–120 h phase (NEPA 76.1% vs. 63.1% aprepitant). As NEPA exhibits long‐lasting efficacy, this study evaluated a prolonged period up to 144 h, beyond the traditional 120 h post‐chemotherapy. In this post‐hoc analysis we explore the comparative efficacy of NEPA versus the aprepitant regimen in the MEC group up to 144 h, while also assessing the impact of risk factors on CINV prevention.

**Methods:**

This was a pragmatic, multicenter, randomized, prospective study. Oral NEPA was administered as a single dose on day 1, while aprepitant was given on days 1–3 + ondansetron on day 1; all patients were to receive dexamethasone on days 1–4. Patients were chemotherapy‐naïve and receiving MEC, with a subset evaluation of those with a risk factor for developing CINV (i.e., female, male <60 years, male ≥60 years who received carboplatin, or male ≥60 years with anxiety). CR rates were compared during the extended overall (0–144 h) phase.

**Results:**

The MEC group included 211 patients; of these 181 were in the risk factor subset. Significantly higher CR rates were seen for NEPA than aprepitant during the extended overall phase for the total MEC group (NEPA 77.1%, aprepitant 57.8%, *p* = 0.003) and also in the subset of patients with CINV risk factors (NEPA 73.9%, aprepitant 56.2%, *p* = 0.012).

**Conclusion:**

A single dose of NEPA, administered on day 1 only, was more effective than a 3‐day aprepitant regimen in preventing CINV for an extended duration in patients receiving MEC and in those with emetic risk factors.

## Graphical abstract

This was a post‐hoc analysis of a pragmatic prospective study exploring the comparative efficacy of NEPA versus an aprepitant regimen in preventing chemotherapy‐induced nausea and vomiting (CINV) in patients receiving moderately emetogenic chemotherapy and in a subset of those with emetic risk factors. This study uniquely assessed complete response (CR: no emesis and no rescue use) rates up to 144 h post‐chemotherapy. Significantly higher CR rates were seen for NEPA than aprepitant during the extended overall phase for all patients receiving MEC (NEPA 77.1%, aprepitant 57.8%, *p* = 0.003) and in those with CINV risk factors (NEPA 73.9%, aprepitant 56.2%, *p* = 0.012).

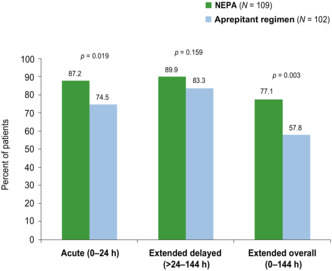

## 1 INTRODUCTION

Concurrent use of multiple antiemetic agents targeting different molecular pathways involved in eliciting chemotherapy‐induced nausea and vomiting (CINV) has transformed supportive care for cancer patients undergoing emetogenic chemotherapy.^1^ In patients receiving highly emetogenic chemotherapy including anthracycline/cyclophosphamide (AC)‐based regimens, evidence‐based antiemetic guidelines^1^, ^2^, ^3^ all consistently recommend co‐administration of a combination regimen consisting of a neurokinin‐1 (NK_1_) receptor antagonist (RA), 5‐hydroxytryptamine‐3 (5‐HT_3_) RA and a corticosteroid, such as dexamethasone (DEX) (± olanzapine).^2^, ^3^, ^4^ However, in patients receiving moderately emetogenic chemotherapy (MEC), guideline recommendations differ. For MEC (aside from carboplatin), the guidelines imply that the evidence is insufficient to recommend an NK_1_ RA “triplet” regimen for all patients and instead endorse a 5‐HT_3_ RA plus DEX “doublet”. Only the National Comprehensive Cancer Network (NCCN) guidelines include in their options a recommendation for an NK_1_ RA‐containing regimen, with the caveat that this is only for those patients with factors that increase their emetic risk or for whom previous 5‐HT_3_ RA plus DEX treatment has failed.^4^

While the guidelines generally make antiemetic recommendations based on the emetogenicity of the chemotherapy, it is well‐established that several patient‐related factors also affect a patient's risk of CINV.^5^, ^6^ A recent comprehensive systematic review of research on these risk factors suggested that antiemetic guidelines and CINV prediction models can be enhanced by considering the findings of the review.^5^ In fact, work is underway by Molassiotis and investigators to explore utilization of a CINV prediction tool in patients receiving MEC to assist clinicians in optimizing antiemetic use for patients with risk factors (NCT04817189).^7^

Because cisplatin is well‐established as the most emetogenic chemotherapeutic, it has served as the standard emetic stimulus for the majority of clinical trials assessing the efficacy of antiemetic agents.^8^, ^9^ In addition, because there is a biphasic time course of emesis following cisplatin, there are two arbitrarily defined phases of CINV following single‐day chemotherapy administration (the acute phase from 0 to 24 h and the delayed phase from 24 to 120 h). Almost all antiemetic studies have therefore evaluated efficacy only up to 120 h after the administration of chemotherapy. However, recent studies have shown that CINV, and especially nausea, can persist beyond day 5 following chemotherapy, suggesting that the time beyond 120 h may be an understudied unmet need in the prevention of CINV.^10^, ^11^

NK_1_ RAs are currently considered interchangeable by antiemetic guidelines. NEPA is the only fixed combination antiemetic agent, uniquely comprising a highly selective NK_1_ RA, oral netupitant, and the clinically^12^ and pharmacologically^13^ distinct 5‐HT_3_ RA, palonosetron. Aside from the simultaneous targeting of two critical antiemetic pathways, it is differentiated from aprepitant with its longer half‐life (80–96 h for netupitant versus 9–13 h for aprepitant).^13^, ^14^ The long elimination half‐lives of both oral netupitant and palonosetron (40 h) could contribute to long‐lasting CINV protection. Prior studies evaluating oral NEPA in the cisplatin HEC setting have shown better prevention of CINV with single‐dose NEPA than a 3‐day aprepitant regimen in the latter part of the delayed phase, specifically during days 3–5 following chemotherapy.^12^ This suggests the efficacy of NEPA is long‐lasting and sustained over time and offers an opportunity to explore the benefits beyond the traditional 120 h time point.

The current head‐to‐head comparative study (NCT03831633) was conducted to evaluate NEPA versus an aprepitant standard‐of‐care (SoC) regimen in real‐world clinical practice in patients receiving either AC or MEC. The primary results of the study assessing efficacy during the traditional 0–120 h time interval were previously published^15^; while non‐inferiority (the primary objective) of NEPA and the aprepitant regimen was shown for the overall population, the overall (0–120 h) complete response (no emesis, no rescue use) rates of 65% for NEPA vs. 54% for aprepitant favored NEPA. Numerically higher complete response rates were also seen for NEPA in the subsets of patients receiving either AC or MEC.

This study uniquely assessed CINV prevention up to 144 h post‐chemotherapy. Herein we describe the comparative efficacy results of NEPA versus the aprepitant regimen beyond the traditional delayed phase in the patients receiving MEC in this study. Additionally, we explore the outcomes during the same time intervals in the subset of MEC patients who presented with patient‐related emetic risk factors.

## 2 METHODS

### 2.1 Study design

This was a pragmatic, multicenter, randomized, single‐cycle, open‐label, parallel group prospective study, conducted at 30 enrolling sites in France between November 2018 and October 2019. The trial protocol was approved by an independent ethics committee, all patients provided written informed consent prior to initiation of any study treatment, and the study was conducted in accordance with recognized international scientific and ethical standards, including but not limited to the International Conference on Harmonization guideline for Good Clinical Practice (ICH GCP) and the Declaration of Helsinki. This trial was registered with ClinicalTrials.gov (NCT03831633).

### 2.2 Patients

Eligible patients in the complete study were adult (≥18 years) males or females, naïve to chemotherapy, and scheduled to receive their first course of AC‐based chemotherapy or non‐AC MEC for treatment of a histologically or cytologically confirmed solid malignant tumor. Patients were required to have an Eastern Cooperative Oncology Group (ECOG) Performance Status of 0–2. Patients were eligible if they were scheduled to receive prevention of CINV with either NEPA or an aprepitant‐based regimen (the SoC in France). Patients were ineligible if they were pregnant or breastfeeding or if they had any hypersensitivity to active substances, excipients or other ingredients of NEPA or aprepitant.

Patients included in these post‐hoc analyses were (1) only those receiving MEC (not including those receiving AC) and (2) also the subset of patients receiving MEC who had a pre‐defined risk factor for developing CINV as per the protocol, i.e.: (1) female, (2) male <60 years, (3) male ≥60 years who received carboplatin, or (4) male ≥60 years with anxiety.

### 2.3 Treatment

Patients were randomly assigned (1:1) to receive either NEPA or aprepitant‐based treatment, both in conjunction with DEX (12 mg) prior to chemotherapy on day 1. NEPA was administered as a single oral capsule (300 mg netupitant, 0.50 mg palonosetron) approximately 1 h prior to chemotherapy on day 1 only, while oral aprepitant (125 mg) was administered approximately 1 h prior to chemotherapy and also once daily (80 mg) on days 2 and 3. Per protocol, intravenous ondansetron (8 mg) was the 5‐HT_3_ RA to be administered in conjunction with aprepitant prior to chemotherapy on day 1. Dexamethasone (8 mg daily) was to be continued on days 2–4 in both groups. Randomization utilized an interactive web response system (IWRS); treatment was initiated on the day of randomization.

### 2.4 Assessments

Demographic data were standardly captured at the screening visit. The presence of anxiety, assessed for the purposes of the risk factor analysis, was evaluated on Day 1 prior to chemotherapy as part of the EQ‐5D health‐related quality of life questionnaire.

From the start of chemotherapy on day 1 until day 7 (0–144 h), each patient completed a diary, capturing emetic episodes, severity of nausea and concomitant medications taken. An emetic episode was defined as any episode of vomiting or retching or combined vomiting and retching. Severity of nausea was evaluated on a daily basis using a 100‐mm horizontal visual analog scale (VAS). The left end of the scale (0 mm) was labeled as ‘no nausea,’ and the right end of the scale (100 mm) was labeled as ‘extremely strong nausea’.

The primary efficacy endpoint in the study was complete response (CR: no emesis and no rescue medication) during the overall (0–120 h) phase following initiation of chemotherapy. In the present post‐hoc analyses of the overall MEC group and in those patients with emetic risk factors, evaluation of complete response was the primary objective. Complete response was assessed during the extended overall phase of 0–144 h and extended delayed (>24–144 h) phase; the previously published acute (0–24 h) phase is included for completeness. Presentation of CR rates during the extended overall phase (0–144 h) relative to the overall phase (0–120 h) (presented in the primary paper^15^) is also included to allow for an assessment of the antiemetic control beyond the traditional period of evaluation. No emesis and no significant nausea were also assessed during these same time intervals. No significant nausea was defined as a VAS score <25 mm. Safety was previously reported in the primary manuscript.

### 2.5 Statistical analysis

The Full Analysis Set (FAS) population was used in these subgroup analyses. This population was defined as all randomized patients who received at least one dose of antiemetic treatment and chemotherapy and had a record of any data post randomization. Following the intent‐to‐treat principle, patients were assigned in the analyses to the group they were initially randomized. It should be noted that the CR rates during the traditional overall (0‐120h) phase presented in the primary paper^15^ utilized the “as treated” (AS) population, per protocol, for the primary study endpoint. The AS population included patients from the FAS population but they were assigned to the treatment group according to the study treatment they actually received.

For the efficacy endpoints, the Pearson's chi‐square test was used to compare proportions in the overall MEC group and separately in the subset of the MEC group with risk factors. For this purpose, the procedure FREQ of SAS version 9.4 was employed.

## 3 RESULTS

### 3.1 Patient population

Of all patients in the overall FAS study population (*n* = 373), a total of 211 patients were included in the MEC group; of these, 181 (86%) comprised the subset of patients with pre‐defined CINV risk factors.

Baseline characteristics were generally similar between the two treatment groups for patients in this MEC group; however, there were more females in the aprepitant group, but more patients receiving carboplatin in the NEPA group (Table 1). Gastric cancer and lung cancer were the most common cancer types and oxaliplatin and carboplatin were the most commonly administered MEC.

**TABLE 1:** Patient baseline characteristics (overall MEC group).

Characteristic	NEPA (*N* = 109)	Aprepitant regimen (*N* = 102)
Gender		
Male	65 (59.6%)	48 (47.1%)
Female	44 (40.4%)	54 (52.9%)
Age (mean ± SD)	65.6 ± 10.56	63.44 ± 10.14
Most common (≥5%) cancer types		
Gastric	34 (31.2%)	36 (35.3%)
Colorectal	20 (18.3%)	16 (15.7%)
Lung/respiratory	19 (17.4%)	12 (11.8%)
Head and neck	7 (6.4%)	8 (7.8%)
Ovarian	6 (5.5%)	9 (8.8%)
Pancreatic	6 (5.5%)	6 (5.9%)
Most common MEC		
Oxaliplatin	56 (51.4%)	57 (55.9%)
Carboplatin	46 (42.2%)	35 (34.3%)
Irinotecan	15 (13.8%)	19 (18.6%)
Risk factor subset		
Female	44 (40.4%)	54 (52.9%)
Male <60 years	18 (16.5%)	18 (17.6%)
Male ≥60 years receiving carboplatin	24 (22.0%)	11 (10.8%)
Male ≥60 years not receiving carboplatin with anxiety	6 (5.5%)	6 (5.9%)

### 3.2 Efficacy

#### 3.2.1 Overall MEC group

**FIGURE 1:**
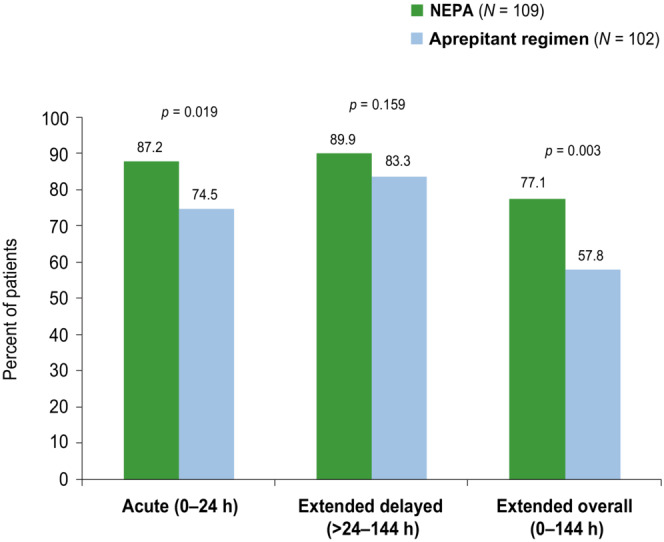
Complete response rates (overall MEC group).

In the overall MEC (non‐AC) population, the complete response rate during the extended overall phase was significantly higher for NEPA (77.1%, *p* = 0.003) compared with the aprepitant regimen (57.8%) (Figure 1). The complete response rate was also significantly higher for NEPA during the acute phase (*p* = 0.019), and higher, but not statistically significant, during the extended delayed phase (*p* = 0.159) (Figure 1).

**FIGURE 2:**
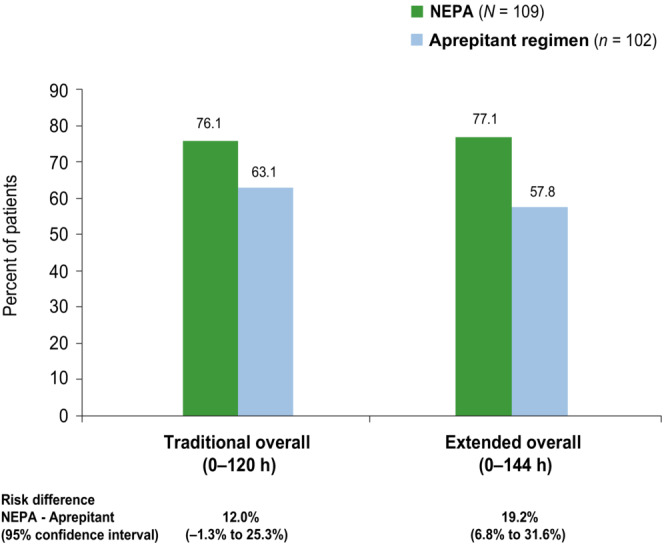
Complete response rates (overall MEC group) during the traditional overall phase (0–120 h) and extended overall phase (0–144 h).

There was an absolute difference of 19.3% in favor of NEPA versus the aprepitant regimen during the extended overall phase compared with 13.0% during the traditional overall phase (Figure 2).

Similar results as that for complete response were seen for no emesis, where significantly more NEPA patients had no emesis during the extended overall (*p* = 0.022) and acute (*p* = 0.009) phases. No significant nausea rates were numerically higher for NEPA during the extended overall and acute phases but not significantly different between the treatment groups (Table 2).

**TABLE 2:** No emesis and no significant nausea rates: overall MEC population and MEC subset with risk factors.

Endpoint	Overall MEC population			MEC subset with risk factors		
	NEPA (*N* = 109)	Aprepitant (*N* = 102)	*p* value	NEPA (*N* = 92)	Aprepitant (*N* = 89)	*p* value
**No emesis**						
Acute (0–24 h)	103 (94.5%)	85 (83.3%)	0.009	87 (94.6%)	73 (82.0%)	0.008
Extended delayed (>24–144 h)	99 (90.8%)	91 (89.2%)	0.696	82 (89.1%)	79 (88.8%)	0.937
Extended overall (0–144 h)	93 (85.3%)	74 (72.5%)	0.022	77 (83.7%)	63 (70.8%)	0.038
**No significant nausea**						
Acute (0–24 h)	91 (88.3%)	81 (84.4%)	0.413	78 (89.7%)	72 (84.7%)	0.331
Extended delayed (>24–144 h)	85 (82.5%)	79 (82.3%)	0.966	69 (79.3%)	68 (80.0%)	0.911
Extended overall (0–144 h)	73 (70.9%)	64 (66.7%)	0.522	60 (69.0%)	55 (64.7%)	0.553

#### 3.2.2 CINV risk factor subset

**FIGURE 3:**
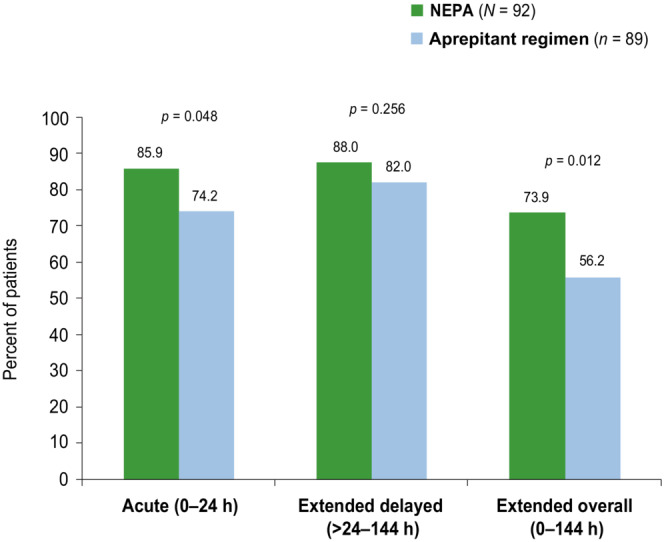
Complete response rates (MEC subgroup with risk factors).

In the subset of patients with risk factors, the complete response rate during the extended overall phase was significantly higher for NEPA (73.9%, *p* = 0.012) compared with the aprepitant regimen (56.2%) (Figure 3). The complete response rate was also significantly higher for NEPA during the acute phase (*p* = 0.048), and numerically higher during the extended delayed phase (*p* = 0.256) (Figure 3). Similar results were seen in this risk factor subset for no emesis and no significant nausea rates as in the overall MEC group (Table 2).

## 4 DISCUSSION

While most studies demonstrating a clear benefit of an NK_1_ RA‐containing triplet over a 5‐HT_3_ RA/DEX doublet have been conducted in the cisplatin‐based highly emetogenic chemotherapy (HEC) setting, there are a few studies which have evaluated NK_1_ RAs in the MEC,^16^ or combined AC/MEC settings.^17^, ^18^ However, antiemetic guidelines have not yet recommended an NK_1_ RA‐containing regimen in the MEC setting. Only NCCN has acknowledged the importance that patient‐related factors may have on elevating the risk for CINV in patients receiving MEC.^4^ Additional studies are needed exploring the benefits of an NK_1_ RA in the MEC setting, particularly in those patients with risk factors. This study is the first head‐to‐head trial comparing one NK_1_ RA versus another in this MEC setting.

In the primary publication of this study Zelek et al. highlight the trend toward better responses with NEPA versus the aprepitant regimen for the primary endpoint of complete response during the traditional 0–120 h overall phase in both the overall study population and in the MEC group.^15^ The 0–120 h complete response rates for NEPA versus the aprepitant regimen were 64.9% vs. 54.1% and 76.1% vs. 63.1% in the overall population and MEC group, respectively.^15^ The presence of factors that increase the risk of experiencing CINV further highlight the possibility that not all NK_1_ RA regimens are the same.^19^ In the subset of patients receiving MEC with emetic risk factors, NEPA was superior to the aprepitant regimen for complete response during the traditional 0–120 h overall phase (73.9% vs. 58.4%, respectively; *p* ≤ 0.05).^19^

This differentiation was also seen in previous analyses in the cisplatin setting in all patients and also in those at the greatest emetic risk receiving the highest doses of cisplatin. Navari et al described the pooled results of three studies which included oral NEPA versus an aprepitant regimen.^12^ A single dose of oral NEPA was superior to a 3‐day aprepitant regimen during the traditional delayed phase (24–120 h) for endpoints of complete response, complete protection (defined as complete response plus no significant nausea), and no significant nausea.^12^ NEPA was also superior to the aprepitant regimen in preventing breakthrough CINV during the individual days 3–5 post‐chemotherapy, suggesting that the efficacy of NEPA is long‐lasting and sustained over time.^12^ In the subset of those at increased emetic risk receiving high‐dose cisplatin, NEPA showed significantly higher complete response and complete protection rates during the delayed phase.^12^

In light of the evidence of this long‐lasting prevention, the current study was uniquely designed to evaluate the comparative efficacy for an extra day beyond that typically assessed (i.e., to 144 h post‐chemotherapy). In this analysis of patients receiving MEC in this study, NEPA showed significantly higher complete response (77.1% vs. 57.8%) and no emesis rates (85.3% vs. 72.5%) than the aprepitant regimen during the extended (0–144 h) overall phase. The high and better response rates for NEPA during the acute phase may have contributed to the significantly better control during the extended overall phase. During the extended delayed phase, the complete response rate was also numerically higher for NEPA (89.9% vs. 83.3%), while no emesis rates were similar for the treatment groups (90.8% vs. 89.2%). Although no significant nausea rates were higher for NEPA than aprepitant during the extended overall phase (70.9% vs. 66.7%) the difference was not statistically significant.

There was an incremental benefit of 6.3% in complete response rates in favor of NEPA during the extended overall phase (a 19.3% absolute difference) relative to the traditional overall 0–120 h phase (a 13.0% absolute difference), again suggesting NEPA may exhibit long‐lasting effects. The efficacy benefit seen with NEPA for the study's primary endpoint of complete response and for preventing emesis during the extended overall phase may be due to the differences in plasma elimination half‐lives of netupitant (80–96 h) versus that for aprepitant (9–13 h).^13^, ^14^

Another study also evaluated antiemetic efficacy beyond the traditional 120 h timepoint, in this case up to 168 h.^20^ This was a non‐inferiority registration study (CONSOLE) conducted in Japan where fosnetupitant (the intravenous (IV) component of IV NEPA) was compared to fosaprepitant (the IV formulation of aprepitant) for preventing CINV in patients receiving cisplatin‐based HEC. Both NK_1_ RAs were administered with palonosetron and dexamethasone. In an exploratory analysis evaluating the comparative efficacy during the extended overall (0–168 h) phase, fosnetupitant had a significantly higher (*p* < 0.05) complete response rate (73.5%) than fosaprepitant (66.9%).^20^

The pre‐defined CINV risk factors of female gender, young age, and anxiety selected in this subset of MEC patients are well‐established for elevating the risk of CINV.^5^, ^19^ In the subset of MEC patients with these identified CINV risk factors, the results were consistent with the overall MEC population for all efficacy endpoints. It is noteworthy that in this risk factor subset, significantly higher response rates were seen for NEPA versus aprepitant during both the previously published traditional overall 0–120 h phase (73.9% vs. 58.4%, *p* < 0.05)^19^ and also during the extended overall 0–144 h phase in the current analysis (73.9% vs. 56.2%).

While comparative cost‐effectiveness was not evaluated in this study, it is noteworthy that studies have been performed showing NEPA to be more cost‐effective than an aprepitant‐based antiemetic regimen, albeit not in a French population.^21^, ^22^

Limitations of this study/analysis include that it was an open‐label design which, although common in pragmatic trials, is susceptible to bias. Another potential limitation is the imbalance seen for baseline characteristics of gender and carboplatin use. In this MEC group, there was a greater proportion of females receiving the aprepitant regimen than NEPA, but also a greater proportion of NEPA patients received carboplatin, a more emetogenic chemotherapy than oxaliplatin and irinotecan. It is possible that these two differences in factors contributing to emetic risk balanced the groups. And finally, as with all post‐hoc analyses, the importance of these findings must be properly considered in the context of their exploratory characteristic.

It is noteworthy that the absolute difference in complete response rates during the extended overall phase was 19% in the total MEC group and 18% in the subset with CINV risk factors. Per the Multinational Association of Supportive Care in Cancer/European Society of Medical Oncology (MASCC/ESMO) guidelines, such large differences (>10%) are clinically meaningful.^3^ Certainly, the results of this study are interesting; however, the clinical relevance of improving CINV control during the extended overall phase in high‐risk patients undergoing MEC should be further evaluated in well‐designed randomized studies.

NEPA is a novel fixed combination antiemetic that simplifies dosing and eliminates the complexity of administering multiple agents and over multiple days (such as is the case with the oral aprepitant regimen), thereby minimizing the potential for non‐compliance with treatment. In this first NK_1_RA comparative analysis of patients receiving MEC and in those with additional CINV risk factors, single‐dose NEPA showed significantly better control of CINV than the aprepitant regimen for an extended duration post‐chemotherapy.

## AUTHOR CONTRIBUTIONS

**Laurent Zelek:** Conceptualization (equal); data curation (equal); investigation (equal); methodology (equal); project administration (equal); resources (equal); supervision (equal); validation (equal); visualization (equal); writing – review and editing (equal). **Rudolph M. Navari:** Validation (equal); visualization (equal); writing – review and editing (equal). **Matti Aapro:** Validation (equal); visualization (equal); writing – review and editing (equal). **Florian Scotté:** Conceptualization (equal); data curation (equal); investigation (equal); methodology (equal); project administration (equal); resources (equal); supervision (equal); validation (equal); visualization (equal); writing – review and editing (equal).

## CONFLICT OF INTEREST STATEMENT

Laurent Zelek reports personal consulting fees as an advisory board member for Vifor during the conduct of the study and personal fees from Novartis, Roche, Lilly, Sandoz and Pfizer, outside the submitted work. Rudolph Navari has nothing to disclose. Matti Aapro reports serving in a consulting/advisory role for Amgen, BMS, Daiichi Sankyo, Fresenius Kabi, G1 Therapeutics, Genomic Health, Helsinn Healthcare, Merck, Merck KGaA, Novartis, Pfizer, Pierre Fabre, Roche, Sandoz, Tesaro, and Vifor Pharma; speakers' bureau at Accord Research, Amgen, Biocon, Dr Reed, Genomic Health, Helsinn Healthcare, Mundipharma, Novartis, Pfizer, Pierre Fabre, Roche, Sandoz, Taiho Pharmaceutical, Tesaro, and Vifor Pharma; and receiving research funding from Helsinn Healthcare, Novartis, Pierre Fabre, and Sandoz. Florian Scotté reports personal consulting fees as an advisory board member for Helsinn Healthcare, Vifor, MSD and Tesaro during the conduct of the study and personal fees from Roche, Amgen, Pfizer, Leo Pharma, Mylan, Mundi Pharma, Clovis Oncology, BMS, Biogaran, Pierre Fabre Oncology, Arrow and GSK outside the submitted work.

## ETHICS STATEMENT

The trial protocol was approved by an independent ethics committee, all patients provided written informed consent prior to initiation of any study treatment, and the study was conducted in accordance with recognized international scientific and ethical standards, including but not limited to the International Conference on Harmonization guideline for Good Clinical Practice (ICH GCP) and the Declaration of Helsinki.

## CLINICAL TRIAL REGISTRATION NUMBER

This trial was registered with ClinicalTrials.gov (NCT03831633).

## Data Availability

Research data are not shared.
